# Callous–Unemotional Traits and Their Association with Neurodevelopmental Disorders: Insights from Gaze Behaviour During Emotion Recognition

**DOI:** 10.3390/children13020303

**Published:** 2026-02-22

**Authors:** Astrid Priscilla Martinez-Cedillo, Christian A. Delaflor Wagner, Lilia Albores-Gallo, Tom Foulsham

**Affiliations:** 1 Department of Psychology, Edge Hill University, Ormskirk L39 4QP, UK; 2 Department of Clinical Research, National Medical Centre 20 de Noviembre, Institute for Social Security and Services for State Workers (ISSSTE), Mexico City 03104, Mexico; 3 Hospital Psiquiatrico Infantil Juan N. Navarro, CONASAMA. Psychiatric Services, Mexico City 14080, Mexico; 4 Department of Psychology, University of Essex, Colchester CO4 3SQ, UK; foulsham@essex.ac.uk

**Keywords:** neurodevelopmental disorders (NDs), attention-deficit/hyperactivity disorder (ADHD), conduct disorder (CD), autism spectrum disorder (ASD), callous–unemotional (CU), eye-tracking, gaze, emotion recognition

## Abstract

**Highlights:**

**What are the main findings?**

- Individuals with CU traits (and those with ASD, ADHD, or CD) show atypical eye-gaze behaviour, especially reduced attention to the eye region, most notably when viewing fearful faces.
- Co-occurrence of CU traits with ASD, ADHD, or CD amplifies avoidance of the eyes during emotional processing, suggesting compounded socioemotional difficulties.

**What are the implications of the main findings?**

- While theories such as amygdala dysfunction, oculomotor disinhibition, and hostile attribution bias explain aspects of gaze behaviour, none fully capture the complexity, highlighting the need to view CU traits as a developmental, cross-disorder construct shaped by environmental factors.

**Abstract:**

Callous–unemotional (CU) traits are characterised by reduced empathy, guilt, and emotional responsiveness, and are strongly linked to atypical socioemotional processing. Eye-tracking research provides a valuable window into these processes by capturing early developing patterns of attention to emotionally salient social cues, particularly facial expressions. This narrative review examines how alterations in gaze behaviour contribute to the emergence of CU traits across neurodevelopmental disorders (NDs), with a focus on autism spectrum disorder (ASD), attention-deficit/hyperactivity disorder (ADHD), and conduct disorder (CD). Across studies, elevated CU traits are associated with reduced fixations on the eye region, most consistently in response to fearful faces. ASD is associated with robust eye avoidance, ADHD with inhibitory and attentional control difficulties during face processing, and CD with atypical gaze allocation to negative emotional expressions such as fear and anger. These patterns appear amplified when CU traits co-occur with NDs. Competing explanatory accounts, including aberrant amygdala functioning, oculomotor disinhibition, and hostile attribution biases, each capture aspects of these patterns but fail to provide a unified explanation. Integrating developmental, neurobiological, and environmental perspectives, we propose that CU traits reflect a transdiagnostic developmental construct shaped by early attentional–emotional mechanisms, rather than a disorder-specific identity.

## 1. Introduction

Callous–unemotional (CU) traits are defined in those who show a pattern of emotional sensitivity including a significant lack of empathy, guilt, and emotional responsiveness [1,2,3]. Eye-tracking research has increasingly been used to explore the early mechanisms underlying these traits, particularly in relation to how individuals attend to and process emotional information. Our question in this review is: How do early developmental processes contribute to the emergence of CU traits, particularly in relation to other neurodevelopmental disorders (NDs)? To answer this question, we will focus on (1) gaze behaviour as a window into early emotional and social processing, and (2) the presence and overlap of CU traits within ASD, ADHD and CD. We do not aim to provide an exhaustive review of CU traits in adulthood, nor do we focus on intervention strategies already established for older individuals. It is particularly important to focus on infancy and early childhood, as this is a period when emotional and behavioural patterns are more malleable and observable [4]. Early identification is crucial, given that persistent CU traits are associated with later antisocial outcomes, including conduct disorder and psychopathy [2].

We begin by examining CU traits and the role of gaze behaviour in uncovering their developmental mechanism. Gaze offers a unique window into initial sensory and emotional processing, and particularly into how individuals with elevated CU traits attend to salient social cues, such as emotional faces, revealing early disruptions in social attention. While CU traits have been most strongly linked with CD, increasing evidence highlights their relevance in ASD and ADHD. These disorders frequently co-occur, share overlapping behavioural profiles, and may involve similar disruptions in sensitivity to social cues [5,6]. By focusing on these early-emerging conditions, we aim to shed light on shared mechanisms that contribute to the developmental pathways of CU traits from infancy onward.

Although anomalies in social and emotional processing are well-documented in ASD and CD, they appear to be less central in ADHD [7,8,9]. Studies on ASD indicate altered social–affective processing, such as difficulties in interpreting facial expressions and emotional cues [10], which can resemble the emotional blunting seen in those with CU traits [11]. Individuals with CD and CU traits often exhibit reduced responsiveness to distress cues, suggesting a potential sensory-processing component [9]. However, ADHD tends to be more associated with non-social sensory processing difficulties, such as hyper or hypo-reactivity to environmental stimuli, rather than deficits in emotional perception [12].

Although CU traits share behavioural features with ASD, CD and ADHD, the underlying mechanisms may differ [11,13]. In ASD, externalising behaviours stem from social communication difficulties, while in CD with CU traits, they reflect a lack of empathy or concern for others [11,14]. ADHD, by contrast, is defined by inattention and hyperactivity rather than emotional insensibility [15]. They also differ in co-occurrence rates: CU traits co-occur with ADHD in approximately 6% of cases, while the prevalence in ASD is estimated between 36 and 51%, and in CD between 21 and 50% [16,17]. NDs typically emerge throughout childhood due to genetically driven atypicality in the brain systems involved in early-stage processing of environmental stimuli [18]. Individuals with an ND are diagnosed when functional impairments become evident, often impacting family life. ASD is usually identified by age 2–3, and is characterised by social communication impairments, repetitive behaviours, and sensory anomalies [15,19]. ADHD is often diagnosed around the age of 6, and is characterised by difficulty concentrating, lack of self-control, and impulsive behaviour [15].

Behaviour problems associated with CU traits increase across childhood and adolescence. Early signs—such as irritability and callousness—can be observed within the first two years of life and can be distinguished from typical variation as early as infancy [20,21,22]. Accurate diagnosis requires interdisciplinary assessment, as CU traits and ND behaviours can resemble typical development. Early identification enables timely intervention, which may reduce the risk of persistent socially challenging behaviours into adulthood [23]. This risk is especially high during adolescence, where CU traits (alone or co-occurring with NDs) are linked to greater aggression and an increase the likelihood of severe, sudden violence [21,24,25]. To better understand the developmental mechanism of CU traits, we must consider how individuals initially process and respond to emotional stimuli. A deeper understanding of disruptive behaviour such as a lack of remorse, emotional dysregulation, irritability, and manipulation—can enhance diagnostic accuracy, improve treatment approaches, and deepen our understanding of the underlying pathology of NDs [1]. One critical aspect of this understanding involves investigating individuals’ initial sensory processing of stimuli present in their surroundings. This helps us determine how individuals identify, process, and perceive salient cues such as emotional faces. Gaze provides a good measure of this initial processing, and there are many observations of changes in gaze and looking behaviour in NDs. Parents and caregivers often report that children with NDs avoid eye contact, though patterns differ depending on the specific condition. For instance, children with ADHD often have difficulty maintaining attention for extended periods particularly during school hours when they are expected to focus on complex cognitive tasks [26]. Similarly, individuals with ASD may not respond when their name is called, often avoid making eye contact, and instead, tend to focus intently on objects for extended periods [27]. In contrast, individuals with CD often exhibit a confrontational attitude [28]. The study of these distinct behavioural patterns has been a significant area of research. However, observations regarding individuals with CU traits remain less clear.

In the following sections, we examine how gaze behaviour, measured using eye-tracking technology, can help describe emotional processing in individuals with elevated CU traits, both with and without co-occurrent NDs. Our focus will specifically be on studies employing eye-tracking technology as we explore subtle differences in gaze patterns. We searched the literature for studies investigating gaze behaviour using eye-tracking methods and assessing CU traits using validated clinical- or questionnaire-based measures, in samples with CU traits either alone or co-occurring with NDs such as ASD, ADHD or CD. Searches were conducted in major electronic databases, including PsychINFO, Pubmed, and Web of Science. We searched the literature up to December 2025 for studies investigating gaze in those with CU traits and co-occurring NDs, using keywords such as: ‘callous–unemotional traits’, ‘eye-tracking’, ‘gaze’, ‘autism’, ‘ADHD’, ‘CD’, and ‘neurodevelopmental disorders’ to identify relevant studies. Only peer-reviewed empirical studies were considered. We also consider whether gaze, as a means of understanding behaviour and responses to emotional stimuli, has a distinctive pattern in those with CU traits and, if so, whether this changes over development. We also consider studies that examine subclinical samples, as these investigations offer valuable insights into the progression of CU traits, their severity, and associations with other NDs. Subclinical samples also provide valuable insights into how these disorders begin to develop, often before individuals reach the criteria for a formal diagnosis [29]. Studies across developmental stages, from early infancy to adolescence, were considered where available. For interested readers, and future systematic efforts, we provide a detailed summary of the sample sizes, eye-tracking paradigms, and clinical assessments used by the authors of the key papers in Table 1. In a recent review, [30] the authors highlighted emotion recognition difficulties in children and adolescents with elevated CU traits. This included a focus on negative emotions and reduced attention to the eye region. Our focus here is different because we will examine, in particular, the co-occurrence of neurodevelopmental conditions such as ADHD and ASD. This, along with the examination of longitudinal development from early infancy, is critical for understanding gaze and emotion across disorders.

However, as detailed in Table 1, the studies included in this body of work are characterised by substantial methodological heterogeneity, which constrains the synthesis and interpretation of findings across disorders. Specifically, there is wide variation in stimulus and task, including preferential looking paradigms in early infancy and static emotional stimuli that manipulate emotional expression and gaze direction. Task demands also vary considerably, from passive viewing to explicit emotion recognition, emotion matching, or cued versus uncued attention to the eye region, resulting in a requirement for different levels of top-down attentional control. In addition, pronounced developmental heterogeneity is evident, with samples spanning infancy, childhood and adolescence, introducing age-related variability in face processing and emotional understanding that may confound associations between gaze patterns and callous–unemotional (CU) features. Finally, the operationalisation of CU traits differs across studies, with multiple instruments used (e.g., APSD, ICU) and both dimensional and categorical approaches, which assess overlapping but non-equivalent constructs. Collectively, these sources of heterogeneity limit the validity of direct cross-study and cross-disorder comparisons, as observed similarities or discrepancies in gaze patterns may reflect methodological differences rather than disorder-specific mechanisms.

## 2. How Does Eye-Tracking Research Reveal the Progression of CU Traits from Infancy to Adulthood?

Recent research highlights the importance of studying infants’ empathy and prosocial behaviour during their critical developmental stage, disruptions in which could be a precursor to CU traits later in life [22,54]. Donohue and others [55] studied infants aged 11 to 20 months through a series of tasks designed to examine whether attention towards the face area was related to infants’ empathic and prosocial skills. These tasks included two prosocial helping tasks (involving balls and blocks), a comforting task, and an emotional eye-tracking task. In prosocial helping tasks, infants watched an experimenter struggle to reach balls or blocks, with prompts like “I can’t reach” and “Can you help?” The trial ended when the infant assisted. In a comforting task, a caregiver pretended to injure her knee, allowing researchers to assess the infants’ emotional responses and comforting behaviours. The eye-tracking study presented a face in the centre of the screen, followed by another face to the left or right, revealing various emotions. The findings showed that infants who paid less attention to negatively emotional faces, especially angry ones, exhibited lower empathy and fewer prosocial behaviours. Their focus on the eye regions also correlated with helping behaviour, indicating that attention to the eyes was more important than overall face attention. Infants who ignored the eye regions of angry, fearful, and sad faces also demonstrated fewer prosocial tendencies.

Research suggests that atypical patterns of empathy and prosocial behaviour are not merely correlated but central features of elevated CU traits [22]. One compelling line of evidence implicates disrupted attention to emotionally salient facial cues, particularly the eyes, as a potential mechanism underpinning these traits. Dadds and others [38] demonstrated that children with elevated CU traits consistently avoided the eye region when viewing fearful faces, instead showing a preference for the mouth, an attentional pattern that may reflect a deeper deficit in processing others’ emotional states. This finding aligns with more recent work by Donohue and others [55], reinforcing the view that reduced looking at the eyes is not incidental but may be foundational to the development of CU traits. Further supporting this claim, other authors [39] found that adolescent males with elevated CU traits made fewer and shorter fixations on the eyes and performed worse in fear recognition tasks, indicating a direct link between gaze behaviour and emotional understanding.

However, not all evidence supports a straightforward relationship. Hartmann and Schwenck [44] assessed children and teenagers in an emotion viewing and categorisation task. In these tasks, the authors tested three free-viewing emotions (anger, fear and sadness). The study found no evidence that eye-preference mediated the relationship between CU traits and deficits in emotion recognition or categorisation. Instead, their results point toward slower processing speed as a more plausible explanatory factor. This raises critical questions about whether reduced eye attention is a cause, consequence, or merely one manifestation of a broader cognitive difference in individuals with CU traits. Adding further complexity, Ivanova-Serokhvostova and others [45] showed that the nature of attentional deficits varies depending on how CU traits are measured. The authors assessed children and early adolescents during an emotion viewing task under conditions in which participants either gazed freely or were instructed to focus on the eyes or mouth. When CU traits were classified using the Child Problematic Traits Inventory (CPTI), participants with elevated CU traits showed reduced attention to the eyes for fear and sadness. However, when CU traits were assessed using the Clinical Assessments of Prosocial Emotions (CAPE), a general attention deficit was observed across all emotions, with increased attention to the mouth area. These inconsistencies underscore the need to refine both conceptual and methodological approaches, rather than viewing gaze aversion as a universal marker of CU traits.

There is other evidence that children with elevated CU traits show reduced sensitivity to facial emotions, fixating less on the eyes and more on the mouth, regardless of the emotion (fear, sad, angry, or happy) or the age of the face (adult or child). This attentional neglect to the eye region may explain their poor performance in identifying facial emotions [41]. Dadds and others [56] examined eye contact in children with elevated CU traits during interactions with their attachment figures—either their mother or father—using behavioural coding from a video rather than eye-tracking technology. Their findings show that children with elevated CU traits lacked eye contact with their attachment figures. Interestingly, fathers of children with elevated CU traits showed a similar pattern, while mothers of children with elevated CU traits did not exhibit this behaviour with their children. Further research by Kyranides and others [47] evaluated adolescents and adults with traits of CU, conduct problems and anxiety during an emotion recognition task, analysing both accuracy and eye-gaze patterns. Instead of static images, videos showing dynamic expressions of anger, fear, happiness, sadness, pain and neutrality were used to better simulate real-world facial interactions. The results revealed that individuals with CU traits (vs anxiety traits) performed the worst in an emotion recognition task and had fewer overall fixations when looking at angry and fearful expressions.

These impairments in emotional processing are significant in the context of antisocial behaviour. Frick and Viding [20] emphasise that CU traits are strongly associated with more severe and chronic antisocial tendencies, distinguishing affected individuals from those with less extreme behavioural issues. Beyond difficulties in emotion recognition, CU traits are linked to cognitive deficits in moral reasoning, and emotional processing, characterised by a lack of empathy and emotional depth, which further distinguishes them from other individuals exhibiting less severe antisocial tendencies. This connection raises important questions about the developmental trajectory of CU traits and their potential role in the onset of psychopathy.

While this review does not primarily focus on Antisocial Personality Disorder (ASPD), understanding its relationship with CU traits remains relevant. CU traits may precede the onset of psychopathy [57]. Although the DSM-5 does not include psychopathy as a formal diagnosis, it considers a psychopathic subtype of ASPD characterised by features such as manipulativeness, deceitfulness, callousness, hostility, irresponsibility, impulsivity, and a propensity for risk-taking. ASPD is defined primarily by a pervasive pattern of disregard for, and violation of, the rights of others, typically beginning in childhood or early adolescence and continuing into adulthood, with a diagnosis requiring evidence of CD before age 15 [15]. While psychopathy cannot be diagnosed in children; CU traits and CD may indicate risk factors. Psychopathy affects 4.5% of adults, with a higher prevalence in males [58,59]. In UK prisons, it is found in 7.7% of men and 1.9% of women [60]. Psychopathy is typically conceptualised as a multidimensional personality disorder comprising affective features (e.g., lack of empathy and guilt) and interpersonal traits (e.g., superficial charm, callous use of others and narcissism; see [58,61,62]. Research on emotion recognition via eye-tracking shows that individuals with psychopathy, both incarcerated and in community samples, exhibit fewer fixations on the eye region compared to their counterparts without psychopathy [42,43]. Incarcerated males with psychopathy focus less on fearful eyes, while also displaying emotion interpretation deficits related to affective traits [40]. In a community study, affective traits predicted reduced attention to pain and embarrassment expressions, though the sample had more females so may have been unrepresentative of psychopathy [46]. Diaz-Vazquez and others [63] systematically reviewed the literature and confirmed that attention biases, particularly reduced gaze to emotionally informative areas like the eyes, play a significant role in the emotion recognition difficulties associated with psychopathic traits in youth. Their findings also emphasise that these deficits are pervasive across various emotional modalities and are modulated by developmental, clinical and contextual factors, reinforcing the importance of early, tailored interventions.

To sum up, research indicates that reduced attention to the eye region is a key feature in individuals with CU traits, and also of psychopathy, affecting emotion recognition and prosocial behaviour. Eye-tracking studies on infants reveal that reduced fixation on emotional faces, especially negative ones, correlates with decreased empathy and helping behaviour. Similarly, children and adolescents with CU traits exhibit reduced sensitivity to facial emotions, preferring the mouth over the eyes, which may contribute to emotion recognition deficits. These impairments extend into adulthood, where psychopathic individuals—both incarcerated and in the community—demonstrate diminished fixations to the eyes, particularly on fearful expressions.

## 3. How Do CU Traits Affect Gaze Patterns in Individuals with ASD?

ASD is a neurodevelopmental condition primarily marked by difficulties in social communication, repetitive behaviours, restricted interests and sensory abnormalities [15,19,64]. The prevalence of ASD stands at around 1%, and notably higher in males than in females [65]. Individuals with ASD often exhibit a tendency to avoid eye contact, a behaviour that has been extensively studied [66,67,68]. This eye avoidance is a key characteristic that helps in recognising and understanding the various manifestations of ASD. Researchers have closely examined this behaviour to gain insights into the broader social and communication challenges faced by individuals on the spectrum [69]. There are many meta-analyses and reviews already focused on summarising findings in ASD, which consistently report reduced attention to socially relevant regions, particularly the eyes and whole-face areas, compared to non-ASD individuals [67,70,71,72,73,74,75,76]. Considering the phenotypic responses of individuals with ASD in face-scanning studies, we question whether elevated CU traits might affect gaze patterns in those with ASD. Our previous discussions have emphasised that individuals with elevated CU traits tend to look less at angry, fearful, and sad faces, particularly at the eyes. Is there something unique about the co-occurrence of ASD and high CU traits? In a study conducted by Carter Leno and others [17], adolescents with ASD watched a video of an actor expressing various emotions, including happiness, sadness, anger, fear, and a neutral expression. The findings revealed that the ASD + CU group (vs. ASD only) had fewer fixations on the actor’s eyes compared to her mouth when viewing fearful faces. A later study by Carter Leno and others [37] investigated emotion recognition under cued and uncued conditions in a subclinical sample, assessing high vs low traits of CU and ASD in children and teenagers. In the cued condition, participants were directed to fixate a cross at the location of the eyes, while in the uncued condition, they did not have any specific guidance. The findings revealed significant differences between individuals with elevated ASD and CU traits. Those exhibiting elevated CU traits had reduced emotion recognition in the uncued condition, but this deficit was overturned when they were cued to the eyes (and higher traits were associated with better fear recognition in this condition). In contrast, individuals with elevated ASD traits experienced difficulties with emotion recognition exclusively in the cued condition.

These results, therefore, suggest that deficits in recognition with CU traits are at least partly due to a failure to spontaneously attend to the eyes. Abnormal eye contact and emotion recognition in those with ASD traits may have a different mechanism since cueing them to the eyes actually made their performance worse. However, we acknowledge that little research has been done on ASD and CU to understand gaze behaviour. Given the heterogeneity of ASD presentation, more work is needed to explore individual differences and the underlying mechanism driving these effects.

## 4. How Do CU Traits Affect Gaze Patterns in Individuals with ADHD?

ADHD is a prevalent neurodevelopmental disorder characterised by inattention, hyperactivity, and impulsivity [15]. The prevalence of ADHD stands at around 8% of children and adolescents worldwide, with a higher prevalence in boys compared to girls [77,78]. The prevalence in adults is lower, sitting between 2% and 5% [77].

Emotion recognition problems are not always seen as a core symptom of ADHD (see prominent theories from [79,80,81]). However, a recent meta-analysis and systematic review by Lievore, Crisi and Mammarella [82] provided comprehensive evidence that children and adolescents with ADHD experience notable difficulties in emotion recognition, particularly in identifying negative emotions such as fear, anger, and sadness. Unlike individuals with ASD, these deficits seem to be emotion-specific rather than global. The same review cites neurophysiological evidence showing reduced gamma activity and altered amygdala responses during emotion processing, indicating atypical neural functioning.

There is distinct and particularly evident gaze behaviour in individuals with ADHD in the context of inattention. These individuals may struggle to maintain eye contact in conversations or school settings to sustain attention to the speaker. This abnormal gaze behaviour in ADHD vs. without ADHD has been widely reported and studied across different ages [83,84,85]. Unlike ASD, where eye-tracking studies aim to understand social impairments, in ADHD, the focus is on core features like inattention, hyperactivity, and impulsivity. For instance, Bucci and others [86] investigated the oculomotor response in postural control among children with ADHD and examined the effects of methylphenidate (MPH) treatment (vs untreated ADHD children). Both groups (treated and untreated) showed poor performance in the tasks. However, the untreated ADHD children showed more considerable impairments in postural control, thus suggesting that MPH may alleviate some of the motor deficits commonly associated with ADHD.

Numerous studies have examined social cueing, building upon the framework of Posner Cueing Tasks [87,88,89,90,91]. This task measures an individuals’ attention toward the gaze direction of others, thereby enhancing our understanding of social attention processes [92]. The results indicate that adolescents with ADHD exhibit difficulties in orienting their gaze towards the cue compared to those without ADHD [87]. Additionally, children with ADHD show quicker eye movement responses than those with ASD [89,90]. These two results—abnormal gaze orienting and quicker responses—seem to reflect problems with inhibition, i.e., the ability to suppress irrelevant stimuli and maintain focus on socially or contextually relevant cues. This is largely tied to impairments in executive functions and attentional control, which are core characteristics of ADHD [93,94]. Additionally, it has also been proposed that inattentive symptoms in adults are associated with fewer microsaccadic responses to fearful expressions [88].

To sum up, individuals with ADHD struggle with gaze control affecting attention. Unlike ASD, ADHD research focuses on inattention and impulsivity. ADHD individuals also have difficulty following gaze cues and exhibit quicker eye movements, reflecting issues with inhibition and attention. Inattentive adults may show reduced eye responses to fearful expressions. What about ADHD with CU traits? To our knowledge, there are no studies looking uniquely at that combination, and one might speculate that the change in sensitivity to gaze cues in ADHD might interact with the reduced attention to the face in CU traits in an interesting way. However, since ADHD and CD often co-occur, we will now examine investigations of gaze in CD next.

## 5. How Do CU Traits Affect Gaze Patterns in Individuals with CD?

CD is defined according to behaviours that infringe upon the rights of others and violate social norms. It typically arises in childhood or adolescence and often co-occurs with ADHD [95]. Individuals with CD may exhibit behaviours such as aggression, bullying, threatening or stealing [96]. The prevalence of CD is estimated at around 8% worldwide, and males are diagnosed more often than females [97]. The co-occurrence of CD with ADHD is well-documented, showing a great overlap between these disorders. Around 30% to 50% of children with ADHD are also diagnosed with CD, while around 40% to 70% of children with CD also have ADHD [98,99]. Early symptoms reported from nine months in infants who later develop a CD include emotional dysregulation, which may be exacerbated by coercive parenting practices [100]. These highlights parenting style, along with genetic predispositions and environmental influences, as potential causal factors in the development of this disorder. As these influences converge, childhood becomes a pivotal stage where the earliest signs of behavioural patterns begin to emerge. It is during this formative period that the foundational manifestations of behaviours take shape, offering a crucial opportunity to observe and understand their roots. These early expressions provide insight into the developmental progression of emotional recognition and identification, shedding light on patterns that, if not addressed, could develop into more socially challenging behaviours. The culmination of these behaviours, CD, has been largely studied in children and adolescents. Billeci and others [35] evaluated children with Disruptive Behaviour Disorder (DBD), which is considered part of CD, in an emotion recognition task (with happiness, sadness, fear, disgust, anger and neutral expressions) while also measuring CU traits. Results suggested that elevated CU traits were associated with fewer fixations and shorter fixation duration on the eyes of sad faces leading to poor sadness recognition. Airdrie and others [31] evaluated CD, and CU traits, in adolescents with ADHD in an emotional recognition task. Their results indicate that individuals with both ADHD + CD exhibit lower accuracy in recognising fear compared to those with ADHD alone or typically developing peers. Specifically, those in the ADHD + CD group are more likely to misinterpret fear as anger. Additionally, both subgroups with ADHD tend to spend less time focusing on the eye region than control participants. This suggests that while challenges in attending to the eye region are prevalent in ADHD, deficits in emotion recognition may be most pronounced in individuals with comorbid CD, particularly among those displaying CU traits. Similarly, Bours and others [36] found fear as a characteristic misrecognised emotion. In their study, adolescents with ASD, CD and the control group were assessed across neutral, fear, happiness, anger and sadness. The ASD and CD groups showed reduced fixation duration to the eyes across emotions and longer time to the first fixation on fearful faces. Elevated CU traits within the CD group were associated with a shorter first fixation duration at the eye region for fearful faces compared to the control group.

Levantini and others [48] assessed children and teenagers with CD and Oppositional Defiant Disorder with high and low CU traits. Results showed that CU traits correlated with reduced first fixation duration to the eyes of negative emotions and the mouth of positive emotions. Parenting environment moderated the link between CU traits and attention to the eyes of negative faces (higher CU traits associated with reduced attention, but only in negative environments). The authors suggested that children with elevated CU traits and poor emotional gaze processing have greater likelihood for severe behavioural outcomes, especially in harsh parenting environments. In another study of Levantini and others [49], DBD children were assessed with elevated and reduced CU traits. CU traits negatively correlated with sadness recognition and fixation on the eyes of sad and disgusted faces. Martin-Key and others [50] assessed adolescents with CD and Typically Developing (TD) Controls in a dynamic and static emotion viewing task. Their results showed that CD adolescents showed poorer recognition of fear and anger, with reduced fixation on the eyes, especially in males. Higher CU traits were linked to better fear recognition in the CD group, contrary to expectations. Martin-Key and others [51] explored how adolescents perceive emotional body postures (static and dynamic) including angry, fearful, and neutral expressions. Their findings revealed that adolescents with CD struggle to recognise emotional body postures, regardless of their CU traits. Interestingly, those with CD spent less time looking to emotionally informative regions, such as the arms. While elevated CU traits were linked to more typical fixation patterns in males with CD, this did not translate into improved recognition performance. These findings suggest that recognition deficits in CD stem from difficulties interpreting emotional cues rather than inhibitory deficits.

Menks and others [52] combined eye-tracking and fMRI to examine functional brain responses and eye gaze patterns in adolescents with CD. They discovered reduced activation in the right anterior insula during emotional face processing compared to typically developing peers, depending on the emotion type. Adolescents with CD also spent less time fixating on the eye region, particularly for neutral and fearful expressions. Notably, this reduced eye fixation time influenced insula activation, highlighting how attentional mechanism shape the brain’s response to emotional stimuli. Additionally, elevated CU traits were associated with longer fixation on the mouth rather than the eyes, hinting at a nuanced role of CU traits in directing attention to specific facial features. Furthermore, Muñoz Centifanti and others [53] tested adolescents from a juvenile detention centre during an emotion viewing task. They showed that youths with high CU traits had less reflexive attention to fearful eye regions. These authors proposed an intervention in which teenagers with elevated CU traits learn to focus on the eyes of individuals displaying fearful expressions. This approach has shown promising results by guiding the young people to direct their gaze towards the eye region of faces during an emotion recognition task. The authors aimed to enhance their ability to recognise fear, thereby addressing the recognition deficits commonly observed in those with elevated CU traits.

To sum up, adolescents with CD, particularly those with co-occurring ADHD and CU traits, consistently show impairments in emotion recognition tasks. These difficulties are especially pronounced for negatively valanced emotions such as fear and anger, which are frequently misinterpreted or under-recognised. Crucially, these impairments are not merely random errors but seem to reflect a pattern of reduced attention to key facial and bodily cues, such as the eyes, mouth, or gestural signals from the arms and hands.

From a cognitive neuroscience perspective, these findings point to disruptions across both low-level sensory processing and higher-level cognitive functions. On the one hand, reduced fixation on emotionally salient areas may reflect deficits in low-level attentional mechanisms (such as impaired bottom-up processing of salient visual features such as eyes), which ordinarily guide the observer’s gaze toward emotionally relevant cues. In this sense, individuals with CD and CU traits may fail to register basic visual information that typically triggers emotional categorisation. On the other hand, even when such cues are perceived, higher-level processes, such as emotional reasoning, social inference, and perspective taking, may also be compromised. This includes difficulties in integrating perceptual input with stored emotional knowledge or in flexibly interpreting ambiguous social signals. CU traits appear to modulate these higher-order cognitive systems. Adolescents with elevated CU traits may show an overall dampened responsiveness to distress cues, possibly reflecting abnormalities in the amygdala and related limbic structures that underpin empathy and moral reasoning, which is discussed in detailed in the next section.

Importantly, ADHD-related attentional variability may further compound these issues. For instance, individuals with co-occurring ADHD and CD may demonstrate inconsistent attention to emotional stimuli, leading to fragmented or incomplete processing of facial and bodily cues. While ADHD may disrupt sustained attention, CU traits may specifically impair emotional engagement, resulting in a distinctive idiosyncratic profile of emotion processing deficits.

Together these findings suggest that emotion recognition impairments in CD are not rooted solely in an inability to interpret emotional meaning but are also shaped by how attention is deployed at both early sensory stages and later interpretive stages. This highlights the importance of multi-level interventions that target both attentional biases (i.e., gaze training, such as seen in recent work [53]) and impairments in emotional understanding (i.e., social cognitive skills training), particularly in individuals with elevated CU traits.

## 6. How Have Theories Accounted for Abnormal Behaviour in NDs/CU?

One possibility is that this is particularly linked to activation of the amygdala, a small, almond-shaped structure in the brain. The amygdala is central to processing emotions, including fear, and is heavily involved in forming memories, making decisions, navigating social interactions and learning [101]. Extensive research in neuropsychiatry has examined the vital function of this brain region in influencing how we interpret information [102]. The amygdala is known for its involvement in emotional processing, helping to evaluate and respond to potential environmental threats [103,104,105]. Research has demonstrated the critical role of the amygdala in predicting the trajectory of an individual’s gaze, particularly in situations of fear [106]. This has led to the concept of an aberrant amygdala as important for theories of emotional development [106,107]. Research suggests that amygdala abnormalities (whether reduced volume in CU traits and ADHD [108,109,110], or enlargement in ASD [111,112]) may influence these gaze patterns. However, inconsistent findings challenge a unified theory of dysfunction across disorders.

Hostile attribution bias (HAB), the tendency to interpret ambiguous social cues as threatening or malevolent, is one potential outcome of amygdala dysfunction [113]. Reduced emotional responsiveness may predispose individuals to assume hostility in others, facilitating aggressive behaviour even in the absence of clear provocation [114]. Thus, amygdala hypoactivation may not only impair emotional learning but also bias interpretations in a hostile direction. Conversely, in individuals with heightened amygdala reactivity such as those with certain presentations of ASD, HAB may stem from an elevated perception of social threat, although this is likely to manifest with different behavioural consequences [115]. In ADHD, however, HAB could arise through a different mechanism, namely, difficulties in executive functioning and emotion regulation rather than heightened threat perception per se. For instance, impairments in inhibitory control and heightened emotional reactivity may amplify impulsive or aggressive responses to ambiguous social cues, even in the absence of a biased threat interpretation [116,117]. These patterns suggest that while amygdala dysfunction may play a shared role in HAB across neurodevelopmental profiles, the direction and impact of this dysfunction vary depending on the broader cognitive and emotional context, including regulatory deficits in ADHD and hyperreactivity in ASD.

An alternative perspective stems from Barkley’s theory of ADHD as an inhibitory disorder rather than an attention deficit [93,94]. Inhibitory dysfunction impairs executive control, contributing to erratic eye movements [83] and difficulty maintaining gaze on socially relevant features [89,90]. While ADHD-related deficits in oculomotor inhibition may explain altered gaze behaviours, it remains unclear why these issues particularly affect responses to fearful expressions. In the future, this interplay between amygdala function and inhibitory control could offers insights into the social processing difficulties seen in ADHD, CU traits, and related NDs that we have discussed here.

## 7. CU Traits with and Without NDs: Who Is at Increased Risk?

So far, we have not discussed the evidence for genetic factors contributing to NDs and CU traits. Developmental psychopathology offers a framework for understanding individual patterns of behavioural maladaptation [20]. Of particular importance for this review, genes and environment will presumably influence both differences in visual attention and variations in ND development [48]. If early differences in visual attention are indicators of CU traits and possible future NDs, then they could be an important endophenotype for work on the genetic origins of these disorders.

According to twin studies, the estimated heritability for elevated traits of CU behaviours ranges from 45% to 67% [118]. Similarly, the heritability of CD is estimated to be between 40% and 70% [119]. In contrast, ADHD has a heritability estimate of 60% to 90% [120], while ASD shows a heritability rate of approximately 90% [121]. However, the environment also plays a crucial role. In truth, each child’s developmental trajectory unfolds due to their attempt to adapt to unique genetic and environmental factors [122]. Within families, genetic and environmental factors are intertwined. Siblings share part of these factors. Large longitudinal studies of infants with a sibling with an ND diagnosis indicate that infant siblings have a higher probability of receiving a diagnosis of ASD, ADHD or CD compared to the ND prevalence in the general population [123]. Despite the high heritability observed in NDs, the quest to identify a singular genetic explanation for these disorders has consistently proven elusive [84,85,118,124,125]. This challenge arises from the co-occurrence of NDs with other disorders and the heterogeneity manifestations that each disorder encompasses [126].

Recent research has offered valuable insights into the looking behaviour of infants at an increased likelihood for NDs, revealing how these behaviours might signal vulnerabilities to specific conditions [32,33,34,127,128]. In a significant study by Wass and others [128], they reported that infants classified as having an increased likelihood for ASD exhibited notably shorter fixation durations compared to their lower likelihood peers. This finding suggests a potential difference in how these infants visually engage with their surroundings. Additionally, Gui and others [127] reported that infants who demonstrated longer fixation durations on faces (alongside other objects) may be at an increased risk for developing ADHD. This highlights the possibility that the patterns of attention infants display could be critical indicators of future behavioural challenges, specifically to socially relevant stimuli. However, research on older individuals diagnosed with ADHD has reported contrary findings (previously discussed), indicating that fixation durations are shorter compared to healthy individuals on socially relevant stimuli [89,90]. Investigating CU traits, Bedford and others [34] examined emotional regulation in children using a static and dynamic emotion recognition task and measuring gaze. CU and ASD traits were assessed using a parent-reported questionnaire. Results showed that elevated CU traits were associated with poorer emotion recognition in static expressions especially for anger and happiness. However, controlling for autistic traits nullified the association between CU traits and static emotion recognition. Autistic traits were associated with emotion recognition deficits across both static and dynamic tasks. This research therefore identifies potential interactions between CU and ASD traits and highlights that motion cues in dynamic stimuli may improve emotion recognition by capturing attention to emotion-relevant facial features.

Recently, Ilyka and others [129] examined mutual gaze (eye contact) in infants aged 4 to 7 months with either a typical or elevated familiar likelihood of ASD and/or ADHD. The study aimed to explore whether mutual gaze influences later social attention and its potential correlation with ASD diagnoses. The researchers analysed data from video-coded parent–infant interactions and eye-tracking tasks. Eye-tracking assessed infants’ face-orienting abilities through a ‘face pop-out’ task. Findings revealed that infants with an elevated likelihood of ASD and/or ADHD engaged in more mutual gaze with parents compared to those with a typical likelihood. Both elevated-likelihood groups (ASD and ADHD) demonstrated higher mutual gaze levels. Notably, increased mutual gaze at 4 to 7 months was associated with reduced face-orienting responses at 8 to 12 months in infants with an elevated likelihood of ASD, particularly those later diagnosed with the condition. The authors concluded that mutual gaze may serve as an early behavioural marker for social challenges in ASD, but not ADHD.

Despite the compelling and consistent evidence from genetic studies supporting the heritability of CU traits [130], it is essential to underscore that genetic predispositions do not operate in a vacuum. Environmental factors, particularly those arising in early childhood, interact with genetic vulnerabilities in shaping developmental trajectories [20]. A growing body of research in developmental psychopathology highlights the influential role of the caregiving environment in modulating risk and resilience in children exhibiting CU traits. Parenting, in particular, is frequently studied as a central environmental factor given its pervasive influence on a child’s emotional, social, and behavioural development [131,132].

Caregiving practices can either buffer or exacerbate the expression of CU traits depending on their quality, consistency, and emotional tone. For instance, harsh, inconsistent, or neglectful parenting practices have been associated with elevated CU traits [133,134], while warm, sensitive, and responsive caregiving has shown potential in mitigating such traits, especially in early childhood [135,136]. These findings suggest that CU traits are not immutable and may be shaped, at least in part, by early relational experiences. Crucially, parental warmth appears to play a particularly protective role. Children who are genetically predisposed to CU traits but are raised in emotionally supportive environments tend to show fewer behavioural problems and more adaptive social functioning than those raised in emotionally cold or punitive contexts [137].

Furthermore, emotional and social skills (domains closely linked to CU traits) are deeply rooted in early parent–child interactions. Empathy, emotion recognition, moral reasoning, and prosocial behaviour all emerge within the context of early social learning, most often mediated by caregivers [138]. Glenn [24] emphasises how deficits in these domains are hallmark features of CU traits and are often preceded or accompanied by disrupted attachment relationships and poor emotion socialisation.

Similarly, broader contextual factors (such as socioeconomic stress, exposure to violence, or unstable caregiving arrangements) may further compound risk [139,140]. These distal influences can affect both the quality of parenting and the child’s overall emotional climate, thus indirectly shaping the expression of CU traits. Importantly, children with elevated CU traits may also evoke more negative responses from caregivers [1], a phenomenon known as evocative gene–environment correlation, further complicating the developmental picture. This bi-directional influence between child temperament and parenting practices highlights the importance of considering dynamic, transactional models in understanding CU trait development [141].

In summary, while genetic factors provide an important foundation for understanding individual differences in CU traits, they do not determine outcomes in a deterministic fashion. The early caregiving environment, especially the emotional quality of parent–child relationships, plays a significant and potentially modifiable role in either reinforcing or attenuating the emergence of CU traits. Recognising the importance of these environmental influences opens critical opportunities for early intervention and prevention efforts targeting parenting practices and emotional socialisation [1,133].

## 8. Conclusions

Understanding gaze behaviour in individuals with CU traits presents a complex challenge, particularly given the overlap with other disorders. We have reviewed key findings from studies examining CU traits in combination with NDs such as ADHD, ASD and CD while also considering and examining the complexities associated with ASPD. Notably, individuals with CU traits frequently struggle to recognise emotions—such as anger and fear—which are critical for successful navigation of social interactions. These difficulties can also be seen in distinctive gaze patterns, characterised by altered fixations on socially salient regions, including the eyes, mouth and arms—areas essential for decoding emotional expressions and engaging in effective communication. Importantly, reduced eye fixation (particularly for fearful expressions) should not be understood as a universal feature of CU traits, but rather as a probabilistic and task-dependent marker that varies across experimental contexts and individual profiles. These gaze abnormalities are unlikely to arise from a single mechanism; rather, they may reflect the interaction of early neurobiological differences, attention control processes, and downstream socio-cognitive biases operating across development, with these mechanisms being differentially weighted across NDs.

This review emphasises the need to better understand the gaze behaviour distinctions within and across ND conditions. While individuals with CU traits show reduced responsiveness to social cues—suggesting a possible emotional processing deficit—this pattern is not consistently observed across all tasks or emotional expressions, and they may still recognise and process a broad range of emotions effectively. Accordingly, differences in gaze (particularly reduced attention to the eye region) appear to be nuanced, context-sensitive, and contingent on task demands rather than uniformly present. In contrast, individuals with ASD typically experience broader impairments in social communication. ADHD is characterised by brief, fleeting glances at socially relevant stimuli, indicative of gaze scanning deficits. CD and ASPD may present more heterogeneous gaze behaviours, often influenced by the presence of co-occurring disorders. From an integrative perspective, similar gaze phenotypes associated with CU traits may therefore emerge via partially distinct developmental pathways across disorders, including differences in limbic responsivity, oculomotor regulation and learned social cognitive biases such as hostile attribution tendencies, further underscoring that gaze patterns linked to CU traits reflect variable expressions rather than a single uniform pathway. Further clarifying these distinctions is essential for refining theoretical models that capture the complex interplay of these traits and conditions.

It is also important to acknowledge that much of the existing literature is based on male-dominant and clinically referred samples, which may limit the generalisability of current conclusions. Sex differences in social attention, emotion processing, and behavioural regulation are well-documented, yet remain underexplored in relation to CU-related gaze behaviour. Similarly, reliance on clinically referred populations may introduce referral and severity biases, potentially overrepresenting more extreme behavioural profiles. These sampling characteristics may partly account for inconsistencies across studies reporting reduced eye fixation in CU traits. Future research should therefore prioritise more diverse, population-based samples to determine the extent to which observed gaze patterns generalise across sex, symptom severity, and community versus clinical contexts.

The research discussed in this review requires detailed measurements taken in a lab environment (although, see Carter Leno and others [37], for an example of a study performed remotely via the internet). The majority of these studies rely on presenting images of single static faces to the participant. Single-image paradigms simplify analysis but strip away essential components of real-world interactions, such as motion, context and reciprocal engagement. Task structure and stimulus type may therefore strongly influence whether reduced eye fixation is observed in individual with CU traits. It may be possible to use dynamic displays of emotion to increase the realism [51], although this can also make conclusions more difficult as they rely on participants looking at the right feature at the right time for the expression to be interpreted. In healthy adults, there is also considerable evidence that observers attend to images and videos of people differently compared to when they are in a face-to-face interaction with a real person (e.g., [142]). These limitations may reduce the ecological validity of findings about human perception and social behaviour from infancy to adulthood especially in a clinical context. Such constraints also limit the ability to disentangle whether observed gaze differences reflect early perceptual salience, attentional control limitations, or higher-order interpretative processes.

To overcome these challenges, recent studies have used video-based and conversational paradigms as laboratory stimuli [143,144,145]. Others have incorporated wearable devices into naturalistic interactions with infants- and caregivers [146]. This shift allows for deeper investigation into how individuals process complex social information in dynamic contexts and is particularly important for testing whether gaze differences associated with CU traits persist across context or emerge selectively under specific task demands. These approaches are particularly well suited to testing integrative developmental models in which early attentional and affective processes shape later social cognition and behavioural outcomes across different neurodevelopmental trajectories.

To advance research in this domain, we strongly advocate for a comprehensive examination of co-occurrence of NDs, with particular emphasis on distinguishing core diagnostic features from associated dimensional traits such as CU characteristics. While CU traits are often studied in the context of CD or antisocial behaviour, there is increasing evidence that they also emerge across a range of NDs, including ASD and ADHD. Future studies should therefore adopt a transdiagnostic perspective to assess whether CU traits represent a distinct developmental phenotype, a set of overlapping risk markers or a cross-disorder construct with context-specific manifestations, including variability in gaze behaviour across emotional and task conditions. Critically, this approach allows for the identification of shared developmental vulnerabilities alongside disorder-specific mechanisms that shape the expression of CU traits. Adopting a transdiagnostic, developmental framework will be essential for determining whether CU traits reflect a distinct neurodevelopmental phenotype, a convergence of multiple risk pathways, or a cross-disorder construct with context-dependent expression.

In parallel, there is a pressing need to address the ecological validity limitations of current experimental paradigms. Laboratory-based eye-tracking studies, while informative, often fail to reflect the complexity of real-world social interactions. Integrating more naturalistic interaction scenarios, including immersive virtual reality environments and wearable gaze-tracking technologies, will enhance the realism of experimental conditions and more accurately capture everyday gaze behaviour. These approaches will be particularly valuable in investigating how individuals with elevated CU traits engage with social cues across different settings and how gaze patterns dynamically interact with emotional salience, attentional control, and social interpretation in real time.

Equally important is the development of integrated, multimodal research methodologies that combine genetic analysis with detailed behavioural assessments. Such approaches will provide a richer, more holistic understanding of how gaze behaviour manifests across varying contexts and evolves over time. Longitudinal studies are especially critical, as they offer insights into the developmental trajectories of NDs in relation to genetic predisposition and environmental influences. Within this framework, gaze behaviour represents a promising intermediate phenotype linking neurobiological vulnerability with later-emerging socio-cognitive characteristics, including CU traits, provided it is conceptualised as variable- and context-dependent rather than a fixed marker.

Lastly, this research must grapple with a fundamental conceptual question: should CU traits be regarded as a distinct identity or as a cross-disorder construct? Developing a clear, evidence-based theoretical framework that situates CU traits within the broader landscape of social cognition and emotional processing is imperative. An integrative developmental model that explicitly links early neural and attentional mechanisms with later cognitive and behavioural outcomes provides a critical step toward resolving this question. Crucially, such a model must also account for developmental timing and environmental modulation, recognising that factors such as age, sex, caregiving context, and exposure to adversity may shape both the emergence and contextual expression of CU-related gaze patterns, and that these influences may interact differently across ASD, ADHD, CD and related conditions. Such a framework will guide the development of targeted interventions, improve differential diagnoses, and ultimately lead to better-tailored support system for individuals with complex neurodevelopmental profiles.

## Figures and Tables

**Table 1:** Summarises the studies discussed that incorporated eye-tracking methodology as part of their methods. Youth Psychopathic Traits Inventory (YPI); Antisocial Process Screening Device (APSD); Child Behaviour Checklist (CBCL); Inventory of Callous Unemotional traits (ICU); Psychopathy Checklist—Revised (PCL-R); Child Problematic Traits Inventory (CPTI); Clinical Assessments of Prosocial Emotions (CAPE); Self-Report Psychopathy Test (SRPT); Emotion Recognition (ER); Emotion Viewing (EV); Emotions Tested (ET); Attention-Deficit/Hyperactivity Disorder (ADHD); Autism Spectrum Disorder (ASD); Callous–Unemotional (CU); Disruptive Behaviour Disorder (DBD); Typically Developing Controls (TDCs); Oppositional Defiant Disorder (ODD) and Conduct Disorder (CD).

Authors	Sample Size	Eye-Tracking Paradigm	CU Measurements
Airdrie et al., 2018 [31]	ADHD group (*n* = 36), ADHD + CD group (*n* = 27), TDC (*n* = 41); adolescents	ER, ET: happiness, anger, fear, sadness, neutral	YPI
Bedford, et al., 2015 [32]	Stratified random sample of 213 participants from a population-based sample of 1233 first-time mothers	Preferential face tracking vs. a red ball at 5 weeks old	APSD CBCL
Bedford, et al., 2017 [33]	Longitudinal study of 206 full-term infants and their families. Data collection from 6-month period. ER at 6 years and CU behaviours at 7 years	No eye-tracker, face-to-face, still-face reunion. Infants and mothers’ gaze episodes as coded manually. Only data from the face-to-face interaction is part of this study	ICU at 7 years old
Bedford et al., 2021 [34]	292 children aged 7 years old	Static ER, ET: angry, happy, sad, scared, neutral Dynamic ER; videos varied in eye-gaze direction (direct vs averted gaze). Children selected the emotion by matching it to one of four static facial expressions	ICU
Billeci et al., 2019 [35]	35 children with DBD and 23 TDC (ages 7–10)	ER, ET: happiness, sadness, fear, disgust, anger, neutral	APSD
Bours et al., 2018 [36]	122 male adolescents (50 ASD, 44 ODD/CD, 28 TDC); ages 12–19 years (M = 15.4, SD = 1.9)	ER, ET: neutral, fear, happiness, anger, sadness	ICU YPI
Carter Leno et al., 2021 [17]	189 adolescents with ASD; a subset (*n* = 46) completed an eye-tracking task	ER, ET: neutral, sad, happy and angry	ICU
Carter Leno et al., 2023 [37]	171 children (99 autistic, 72 TDC, ages 10–16)	ER with cued (eye cue) and uncued conditions ET: fear, anger, happiness, sadness, surprise	ICU
Dadds et al., 2006 [38]	Two studies with 33 and 65 boys (ages 8–15)	EV, and categorisation under free-gaze, eye-gaze, and mouth-gaze conditions	APSD
Dadds et al., 2008 [39]	100 adolescent males (mean age 12.4 years)	EV and categorisation	APSD
Dargis et al., 2018 [40]	98 offenders (divided into low, intermediate, and high psychopathy groups)	Two eye-tracking tasks: ER and EV	PCL-R
Demetriou & Fanti, 2022 [41]	59 children (mean age = 6.35 years)	Static images of children and adults. ET: fear, sadness, anger, and happiness	CPTI
Gehrer et al., 2019 [42]	30 participants (psychopathic and non-psychopathic offenders)	Emotion and gender categorisation. ET: angry, disgusted, fearful, happy, neutral, sad, surprised	PCL-R
Gillespie et al., 2015 [43]	38 adult male non-offenders (ages 19–39)	EV, ET: angry, disgust, fear, happy, sad and surprise	Levenson SRPT
Hartmann & Schwenck, 2020 [44]	92 boys (7–12 years, M = 9.00, SD = 1.29)	EV, ET: happy, sad, fearful, disgusted, angry, neutral	APSD
Ivanova-Serokhvostova et al., (2024) [45]	52 boys, M = 10.29, SD = 2.06	EV, free gaze, eye gaze, and mouth gaze conditions. ET: happiness, sadness, anger, disgust, fear, neutral	CPTI, ICU, CAPE interview.
Kaseweter et al., 2020 [46]	138 undergraduates (23 males, 115 females)	EV, ET: neutral vs. distress expressions: fear, pain, embarrassment, startle, sadness	SRPT
Kyranides et al., 2020 [47]	80 adults (18–21 years)	EV, ET: anger, fear, happiness, sadness, pain, neutral. A visual probe was used to direct attention to different areas of the face (forehead, eyes, mouth)	ICU
Levantini et al., 2022 [48]	92 boys (7–12 years old). CD (N = 12) and ODD (N = 80)	EV, ET: happy, sad, fearful, disgusted, angry, neutral	APSD
Levantini et al., 2023 [49]	116 children, mean age 9.00 (SD = 1.29), with a DBD	ER, ET: happiness, sadness, anger, disgust, fear, neutral	APSD
Martin-Key et al., 2018 [50]	Adolescents with CD and TDC	Dynamic and static EV. ET: anger, sadness, fear, happiness, surprise, disgust, and neutral expressions	ICU
Martin-Key et al., 2021 [51]	96 adolescents (45 with CD, 51 TDC)	Body posture categorisation (static and dynamic)	ICU
Menks et al., 2021 [52]	58 adolescents (23 CD, 35 TDC); ages 14–19 years	EV, ET: neutral, anger, fear	YPI
Munoz-Centifanti et al., 2021 [53]	34 participants (29 male) from a juvenile detention centre, aged 14–17	EV, ET: fear, anger, happiness, neutral	ICU

## Data Availability

No new data were created or analysed in this study.

## Abbreviations

The following abbreviations are used in this manuscript:

ADHD	Attention-Deficit/Hyperactivity Disorder
APSD	Antisocial Process Screening Device
ASD	Autism Spectrum Disorder
CAPE	Clinical Assessments of Prosocial Emotions
CBCL	Child Behaviour Checklist
CD	Conduct Disorder
CPTI	Child Problematic Traits Inventory
CU	Callous–Unemotional
DBD	Disruptive Behaviour Disorder
ER	Emotion Recognition
ET	Emotions Tested
EV	Emotion Viewing
HAB	Hostile Attribution Bias
ICU	Inventory of Callous Unemotional traits
ODD	Oppositional Defiant Disorder
PCL-R	Psychopathy Checklist—Revised
SRPT	Self-Report Psychopathy Test
TDC	Typically Developing Controls
YPI	Youth Psychopathic Traits Inventory
